# OTVLD-Net: An Omni-Dimensional Dynamic Convolution-Transformer Network for Lane Detection

**DOI:** 10.3390/s25175475

**Published:** 2025-09-03

**Authors:** Yunhao Wu, Ziyao Zhang, Haifeng Chen, Li Jian

**Affiliations:** 1 College of Electronic Information and Artificial Intelligence, Shaanxi University of Science and Technology, Xi’an 710021, China; 221611034@sust.edu.cn (Y.W.); chenhaifeng@sust.edu.cn (H.C.); 2 School of Physics, Peking University, Beijing 100871, China; 1901110194@pku.edu.cn; 3 State Key Laboratory of Artificial Microstructure and Mesoscopic Physics, Beijing 100871, China

**Keywords:** lane detection, Vision Transformer, feature extraction, vanishing point detection

## Abstract

With the vigorous development of deep learning technology, lane detection tasks have achieved phased results. However, existing lane detection models do not consider the unique geometric and visual features of lanes when dealing with some challenging scenarios, resulting in many difficulties and limitations. To this end, we propose a lane detection network based on full-dimensional convolutional Transformer (OTVLD-Net) to improve the adaptability of the model under extreme road conditions and better handle complex lane topology. In order to extract richer contextual features, we designed ODVT-Net, which uses full-dimensional dynamic convolution combined with improved feature flip fusion layer and non-local network layer, and aggregates lane symmetry features by utilizing the horizontal symmetry of lanes. A feature weight generation mechanism based on Transformer is designed, and a cross-attention mechanism between feature maps and lane requests is added in the decoding stage to enable the network to aggregate global feature information. At the same time, a vanishing point detection module is introduced, and a joint weighted loss function is designed to be trained in coordination with the lane detection task to improve the generalization ability of the lane detection model. Experimental results on the OpenLane and CurveLanes datasets show that the detection effect of the OTVLD-Net model has reached the current advanced level. In particular, the accuracy on the OpenLane dataset is 6.4% higher than the F1 score of the second-ranked model, and the average performance in different challenging scenarios is also improved by 8.9%. At the same time, when ResNet-18 is used as the template feature extraction network, the model achieves a speed of 103FPS and a computing power of 14.2 GFlops, achieving good performance while ensuring real-time performance.

## 1. Introduction

With the increasing advancement of autonomous driving technology, lane line detection, a critical component of vehicular environmental perception, is widely utilized in intelligent driver-assistance systems (IDAS) such as lane keeping assist, lane departure warning, blind spot monitoring, adaptive cruise control, and forward collision warning. However, robust lane line detection in complex environments remains highly challenging. The inherent structural characteristics of lane lines their slender morphology, potential for bends, merges, and bifurcations, small footprint, and sparse pixel distribution make them inherently more difficult to identify than conventional objects. Furthermore, actual driving scenarios are complicated by numerous uncontrollable external factors, including adverse weather, extreme illumination, traffic interferences, and abnormal markings.

Early research in lane detection primarily relied on fundamental image features such as color [1] and edge information [2]. Although traditional methods based on handcrafted features offer straightforward implementation and rapid detection speeds, they exhibit limited robustness and poor interference resistance in complex environments. Consequently, these approaches often struggle to meet the high-precision requirements of autonomous driving systems for lane detection. Recent advances in deep neural networks, coupled with the availability of large-scale annotated datasets, have provided powerful new avenues for addressing this problem. Lane detection methods based on pixel-wise segmentation identify lane markings through pixel-level segmentation. Subsequent post-processing techniques—such as clustering and curve fitting—are then applied to derive parametric representations of the lane lines. These approaches can be categorized into semantic segmentation and instance segmentation based on their segmentation output format. Semantic segmentation methods first perform binary segmentation to extract lane contours, followed by clustering algorithms to group lane pixels and distinguish individual lane instances. A key distinction between these methodologies primarily lies in their employed clustering algorithms. For instance, VPGNet [3] employs a density-based clustering algorithm. LaneAF [4] introduces a direction-aware clustering technique utilizing horizontal and vertical affine fields to constrain the spatial arrangement of adjacent lane points, thereby enhancing the interpretability of the clustering process. In contrast, instance segmentation-based methods directly predict segmentation maps for individual lane instances, deriving precise lane geometry through subsequent curve fitting. For example, ConvLSTM [5] leverages temporal information from consecutive frames by integrating a hybrid convolutional-recurrent neural network architecture, further improving lane segmentation accuracy. While segmentation-based approaches offer conceptually straightforward design, the computational resources and inference time required for dense pixel-wise prediction impose non-trivial overhead.

Inspired by rectangular anchor boxes in object detection, researchers introduced dense linear anchors as structural priors for lane detection. These methods predict lane point offsets relative to the predefined linear anchors, which span diverse image regions to accommodate lanes with varying spatial distributions. This design facilitates feature extraction by providing dedicated reference structures. LineCNN [6] pioneered this paradigm shift, enumerating potential lane origins at image boundaries to generate predictions rather than explicitly sampling features via linear anchors. Conversely, PointLaneNet [7] and CurveLanesNAS [8] employ vertical linear anchors to associate each feature map cell with ground truth lane annotations, subsequently regressing positional offsets relative to these anchors. Despite their efficacy, the fixed geometry and predetermined placement of linear anchors constrain adaptability to lanes of arbitrary shapes and orientations, potentially resulting in suboptimal feature sampling.

Parameter prediction-based lane detection methods represent lane markings as parameterized curves and directly regress the corresponding curve parameters via end-to-end network architectures. Bert et al. [9] proposed a differentiable least squares fitting module to optimize lane curve parameters. Compared to the conventional two-stage segmentation-and-fitting pipeline, this end-to-end paradigm enhances stability during model fitting and yields superior performance and interpretability. To address complex lane geometries, PRNet [10] employs piecewise-defined polynomials with distinct coefficients to represent lane structures. LSTR [11] utilizes a hybrid convolutional Transformer architecture to capture global contextual information for lanes and directly regresses lane parameters. However, LSTR’s curve formulation, designed around specific vehicle camera parameters, exhibits high complexity, complicating model training.

In summary, identifying lane lines by pixel-by-pixel classification requires high-resolution feature maps, which leads to a large number of model parameters and high inference latency, making it difficult to meet the real-time requirements of the vehicle system. The clustering algorithm required is prone to failure due to pixel breakage in occlusion or intersection scenes, resulting in lane line merging or breakage. Lane points are sampled by predefined anchor lines and offsets are predicted. The sampling points are unevenly distributed in curved scenes, resulting in curvature estimation deviation. Horizontal anchors are difficult to adapt to steep curves, and high-density anchors improve coverage but increase the amount of calculation. Low-density anchors may miss sparse lane lines. The parameterized curve prediction of direct regression lane lines is insufficiently aware of local slender structures, and the regression error of curve edge points is large. At the same time, it is difficult to strike a balance between the number of model parameters and feature extraction accuracy.

Based on the preceding analysis, we propose ODVT-Net, a novel lane detection model integrating omni-dimensional dynamic convolution with Vision Transformer. To address the limited representational capacity of conventional deep learning models for lane detection, we introduce Omni-Dimensional Dynamic Non-local Fusion Network as the core feature extraction module. Concurrently, recognizing the critical need for robust spatial relationships and global context in lane detection, we incorporate a Transformer-based feature weighting mechanism and a vanishing point detection module. This design enables ODVT-Net to capture rich global information across both spatial and channel dimensions, significantly enhancing the model global perception capability. Furthermore, ODVT-Net achieves end-to-end pixel-level lane detection and effectively adapts to complex topological structures, including bifurcations and discontinuities. The principal contributions of this work are four fold:

(1) In order to extract features with richer contextual information in the feature extraction stage and to improve the adaptive ability of the model under extreme conditions, this paper proposes a full-dimensional dynamic non-local feature extraction module to extract features from the input images. The Omni-Dimensional Dynamic Convolution (ODConv) is used to make the model dynamically adapt to the inputs of different types of complex situations of lane lines, and an improved Non-local network layer is incorporated to help the model capture the long-range dependencies of spatial dimensions, and a Feature Flip Fusion Level is also incorporated to utilize the horizontal symmetry of lane lines, aggregating features of lane symmetry.
(2) In order to aggregate global features, a Transformer-based feature weight generation mechanism is designed, which utilizes the self-attention mechanism of the Transformer encoder while adding a cross-attention mechanism between the feature map and the lane request at the decoder stage, so that the network both aggregates the global feature information while intuitively capturing the correlation between the feature sequences and the a priori sequences of the lane lines, to reducing the complexity of the network.
(3) To further enhance the model’s global perception capability, we introduce a vanishing point detection auxiliary task following feature extraction. This module undergoes joint optimization with the primary lane detection task through shared weight parameters. This design exploits vanishing point localization as a global positional prior while concurrently enriching extracted features and improving model generalization.
(4) To enhance detection performance for complex lane topologies, our method directly predicts pixel-level lane representations and employs bipartite matching against ground truth annotations. The final loss function combines the bipartite matching loss with the vanishing point detection loss in a weighted formulation.

The structure of the rest of this paper is described as follows: Section 2 deeply explores the existing deep learning-based lane detection methods, including the technical ideas based on semantic segmentation, object detection, and parameter prediction. Section 3 describes the lane detection model of full-dimensional dynamic convolution-VIT and the design details of its main modules. Section 4 describes our comparative experiments and ablation experiment results. Finally, Section 5 summarizes our contributions.

## 2. Related Work

### 2.1. Semantic Segmentation Based Methods

Lane line detection methods based on semantic segmentation, such as the Spatial Convolutional Neural Network SpatialCNN [12], which extends the traditional deep layer-by-layer convolution to slice-by-slice convolution in feature mapping to realize message passing between pixels in rows and columns in the layer; structural association-based lane line detection method SAD [13], which is applicable to the accurate detection and tracking of lane lines in automated vehicle systems; group channel local connection-based lane line detection method GCLNet [14], which is applicable to the efficient and accurate detection and tracking of lane lines in automated vehicle systems; and CurveLanesNAS [8], which is based on neural structure search for lane line detection. LaneNet [15] was proposed by the team of Neven et al. Similarly to the clustering method Deep Clustering [16], LaneNet deploys two decoders for segmentation and instance embedding, respectively, which enable it to finish clustering lane lines in the main body of the model without the need of tedious post-processing tasks. In previous segmentation-based lane line detection tasks, such as CooNet [17] and SCNN [18], the maximum number of lane lines is rigidly limited to four, which weakens the flexibility of lane line detection, while LaneNet is able to adaptively detect different numbers of lane lines according to different scenarios. Wang et al. proposed the FENET [19] model inspired by the concentration of human drivers and utilized focus sampling and partial field of view evaluation. focus sampling and partial field of view evaluation to emphasize important long-distance details. GroupLane [20] first applied the row classification strategy to 3D lane detection, performed row classification in the bird’s-eye view (BEV) space, and designed a dual detection head group to process lanes in different directions, respectively, solving the problem that traditional methods only support vertical lanes. Han et al. [20] proposed a new detection head structure that processes lane geometry information and environmental information (discrete key point height regression), respectively, regards 2D lanes as projections of 3D lanes in perspective space, and achieves unified representation through camera parameters, effectively combining integrity and local flexibility.

Early segmentation-based methods typically preset a fixed maximum number of lane lines and were unable to flexibly adapt to changes in the number of lane lines in actual road scenarios. Standard segmentation methods work in perspective image space and are primarily designed for vertical lane lines, making it difficult to effectively model the three-dimensional geometric information of lanes. They also perform poorly for detecting curved lanes, horizontal lanes, or other non-vertical lanes. More importantly, in complex scenarios (such as occlusion, lighting changes, and road wear) or when focusing on distant, small lane lines, the model may struggle to capture sufficiently clear and robust features.

### 2.2. Object Detection Based Methods

Target-based detection methods, such as PointLaneNet [7], a point-based lane line detection network for lane line identification and tracking by detecting lane line points in images; LineCNN [6], a line-based convolutional neural network method for lane line detection by detecting line segments in images; and IntRA-KD [21], a regional affinity kernel density-based lane line detection method for lane line detection and tracking by capturing correlations between different regions in images; UFLDV2 [22], a lane line detection method that integrates feature learning and detection to achieve accurate lane line detection and tracking by jointly learning image features and detector parameters; SGNet [23], a semantic grouping-based lane line detection method that improves lane line detection accuracy by grouping pixels in an image into semantic regions; and CondLaneNet [8], a conditional network based lane line detection method that improves the accuracy and robustness of lane line detection by introducing conditional information, first generates a set of predefined anchor lines, inputs them into a deep learning model to extract the anchor features, calculates the offset between the lane lines and the anchor lines in the image, and finally regresses them back to the predefined anchor lines to obtain the lane line prediction results. DecoupleLane [20] proposes a new detection head structure, which processes lane geometry information (third-order polynomial curve modeling in BEV space) and environmental information (height regression of discrete key points), respectively, and regards 2D lanes as projections of 3D lanes in perspective space, achieving unified representation through camera parameters, effectively combining integrity and local flexibility. LaneCorrect [24] proposes a completely unlabeled lane detection framework, which extracts candidate lane points from the ground point cloud through threshold segmentation based on the high reflectivity difference in special paint on lane markings in point clouds, aggregates candidate points into 3D lane instances, and projects them to 2D images to generate noisy pseudo-labels. From pseudo-label generation to final detection, no manual labeling is required, which greatly reduces the labeling cost. Compared with the segmentation-based lane line detection method, the target detection-based method can accomplish the lane line detection task more quickly due to the use of anchor lines that incorporate the a priori knowledge of the lane line topology. And it is more resistant to interference. However, when encountering special lane line representations such as bifurcations, disconnections, or bends, this approach performs poorly despite its ability to simplify the modeling process.

Although lane detection methods based on target detection have shown advantages in detection speed, topology preservation, and anti-interference ability by introducing prior knowledge such as anchor lines, regional relationships, conditional information, or geometric modeling, predefined models or anchor lines are difficult to flexibly adapt to these irregular or geometric structures that exceed the preset pattern; methods such as DecoupleLane, which treat two-dimensional lanes as three-dimensional projections, are highly dependent on accurate camera internal and external parameters for perspective conversion. Camera calibration errors or dynamic changes will directly affect the accuracy of the detection results; LaneCorrect, a method that uses point clouds to generate unsupervised pseudo-labels, has its detection results limited by the noise generated by the point cloud segmentation and aggregation steps. The inaccuracy of threshold segmentation and the error in aggregating point clouds into three-dimensional instances will cause the pseudo-labels projected onto the image to contain noise, which in turn affects the effect of the final supervised training.

### 2.3. Parameter Prediction-Based Methods

Parameter-based prediction methods, such as PolylaneNet [25], LSTR [11], BezierLaneNet [26], etc., are different from the aforementioned point-based prediction methods, in that parameter prediction methods directly output parameter lines represented by curve equations, which makes the model more end-to-end, reduces the complexity of the model, and makes it easy to obtain a lightweight model. However, the performance of parametric prediction-based lane line detection methods is often not as good as that of point-based methods because modeling lane lines as parametric curves restricts the degree of freedom of the lane line points, and is not compatible with special lane line representations such as irregularities and bifurcations, etc. The PolyLaneNet method was proposed by Lucas Tabelini et al. This model is a convolutional neural network for end-to-end lane line detection estimation and outputs polynomials representing each lane marker in the image, as well as domain lane polynomials and confidence scores for each lane, but its simple network structure and polynomial parameter curves are insufficient to cope with the complex lane line topology, which seriously affects its network performance. The team of Ma et al. proposed the BezierLaneNet method, and unlike the above methods, the model proposes to use a third order. Bessel curves to fit lane lines, and also proposed and used a deformation convolution-based feature flip fusion module in order to fuse the symmetric properties of the lanes in the driving scenario into the features.

Lane detection methods based on parameter prediction offer advantages in terms of model lightweightness and end-to-end deployment. However, parameterized methods force lane lines to conform to a predefined curve equation, limiting the degrees of freedom of lane points and resulting in overly strong geometric constraints. Compared with methods based on object detection, parameterized models often struggle to achieve comparable detection accuracy. The fundamental reason is that the curve equation requires a finite number of parameters to summarize the global shape, while local details of lane lines (such as worn markings and shadow interference) are easily ignored during smoothing, resulting in keypoint offsets. Slight deviations in curve parameters can significantly distort the predicted lane shape, making model training more dependent on fine-tuning parameters and increasing the difficulty of optimization.

## 3. Method

### 3.1. OTVPA-Net Framework

**Figure 1:**
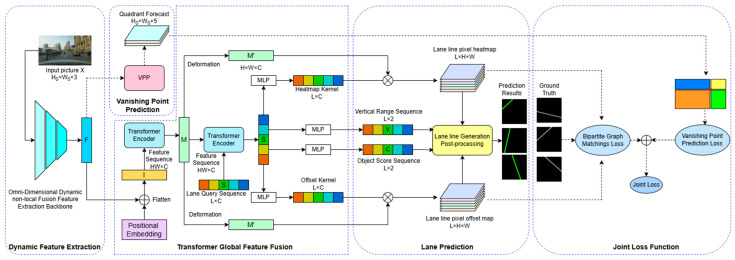
OTVLD-Net network structure.

The network architecture of OTVLD-Net consists of the dynamic feature extraction network ODVT-Net, the vanishing point detection module, the Transformer global feature aggregation module ODVT-Net, the lane detection module and the joint loss module, as shown in Figure 1. The model consists of a sequence of modules that process information in different ways:

(1) Dynamic feature extraction module: We propose a omni-dimensional dynamic non-local fusion feature extraction network, which performs dynamic convolution on the feature map after fusing the lane line symmetry features, and captures the global view of the feature map in the spatial dimension through the non-local layer.
(2) Vanishing point detection module: Use the feature map output in the feature extraction stage to detect vanishing points and generate a vanishing point quadrant prediction map.
(3) Transformer global feature aggregation module: The Transformer encoder is used to perform global feature aggregation on the input feature map in the channel dimension, and the Transformer decoder is used to calculate the cross attention between the feature sequence and the lane request sequence to further expand the global field of view of the feature sequence.
(4) Lane detection module: Utilizes a series of outputs with global feature information extracted by the previous module to perform pixel-level detection of lane lines.
(5) Joint loss module: Calculates the weighted loss of vanishing point detection and lane line matching, and the model is trained based on this loss.

### 3.2. Omni-Dimensional Dynamic Non-Local Fusion Feature Extraction Network ODVT-Net

**Figure 2:**
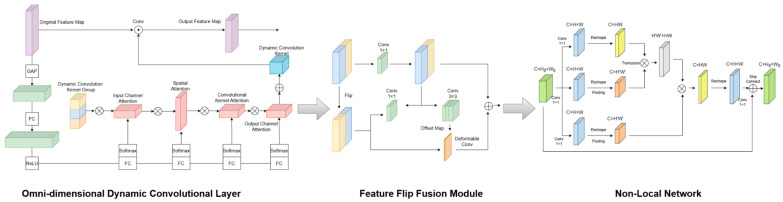
ODVT-Net network structure.

A single CNN network is limited by the narrow receptive field of CNNs and the limitations of the universal convolution kernel. The feature representations it generates are often simple and limited. This fixed feature extraction mechanism limits the model’s ability to adapt to complex scenes. Therefore, this paper designs a full-dimensional dynamic non-local fusion feature extraction network ODVT-Net, whose main structure is shown in Figure 2. First, ODVT-Net replaces the convolution operations of the first two layers of residual blocks in the ResNet model with full-dimensional dynamic convolution operations to form a full-dimensional dynamic convolution layer. This operation enables the model to dynamically adapt to lane line images in different scenarios; secondly, ODVT-Net introduces a feature flip fusion module to introduce the horizontal symmetry of lane lines to the model; finally, ODVT-Net replaces the convolution operations of the last two layers of residual blocks in the ResNet model with improved Non-local Blocks to give the model a global vision in the spatial dimension.

The omni-dimensional dynamic convolution operation (Figure 2, left) uses a new multi-dimensional attention mechanism and parallel strategy. ODConv dynamically learns the attention of the convolution kernel along the four dimensions of the kernel space in the convolution layer, thereby better capturing the feature information of the input data, allowing the network to better adapt to different input data and improve the perception and generalization capabilities of the model. At the same time, there is no great sacrifice in the amount of model calculation. The dynamic convolution operation can be regarded as a dynamic perceptron $y=g({\tilde{W}}^{T}x)+b$, and its calculation process is as follows:

(1) $$y=g({\tilde{W}}^{T}x)+b$$

(2) $$\tilde{W}=\sum_{k=1}^{K}\pi_{k}(x){\tilde{W}}_{k}$$

(3) $$0\leq \pi_{k}\leq 1,\sum_{k=1}^{K}\pi_{k}(x)=1$$

where $\tilde{W}$, b, g represent weights, biases, and activation functions, respectively, and $\pi_{k}(x)$ represents the attention weight. The attention weight is not fixed, but changes with the input. Therefore, compared with static convolution, dynamic convolution has stronger feature expression ability.The additional computation it brings is much less than the convolution itself:

(4) $$O({\tilde{W}}^{T}x+b)\gg O(\sum \pi_{k}{\tilde{W}}_{k})+O(\pi (x))$$

Therefore, applying ODConv to lane line detection models can improve performance without destroying their real-time requirements.

Lane lines on the road often have horizontal symmetry, that is, the lane lines are symmetrically distributed on both sides of the lane, and in most scenarios, the shapes of two symmetrical lane lines are similar. Inspired by the BezierLaneNet [27], ODVT-Net adds a feature flip fusion module (Figure 2, middle) to enable the model to consider the horizontal symmetry characteristics of lane lines during reasoning and provide a reference for symmetrical lane lines for the detection of single lane lines. Considering that the lane lines in the camera-captured image may not be aligned, for example, image angle rotation, vehicle turning, unpaired lane lines, etc., 3 × 3 deformable convolution is used in the convolution layer of the flipped feature map, and the model learns the offset features to make residual connections with the original feature map. Thanks to the fact that OTVLD-Net outputs pixel-level predictions of lane lines in the lane detection task, the positioning of pixel points can build a more accurate spatial feature map, which will support the accurate fusion between flipped features in the feature flip fusion module, help the model better adapt to different road conditions, and effectively improve the performance of the model in complex road environments.

In order to obtain a global view of the spatial dimension at this stage, ODVT-Net focuses on the extraction of non-local information in the spatial dimension in the height H and width W directions. Unlike the classic Non-Local Block, the input feature map size in the Non-Local Block of ODVT-Net (Figure 2, right) is modified to C×H×W, making it more suitable for image detection and focusing on the global information extraction in the spatial dimension. At the same time, in order to reduce the amount of model calculation and not destroy the real-time requirements in the lane detection model, after the two 1 × 1 convolutions of the Non-Local Block, the spatial dimension of the feature map is sampled with a maximum pooling of 2 steps. The impact on network performance is within an acceptable range, but the amount of calculation is only 1/4 of the original. By introducing the Non-Local Block, ODVT-Net can more easily capture long-distance dependencies in the spatial dimension, and at the same time, with the Transformer global feature aggregation, it focuses on the attention extraction in the channel dimension, so that the OTVLD-Net model can obtain a more comprehensive global view. The model will be more suitable for handling lane detection tasks in complex scenes, improving the accuracy and robustness of detection.

### 3.3. Vanishing Point Prediction Module

Adverse conditions—including inclement weather, poor illumination, and occlusions—degrade lane visibility. To address this, OTVLD-Net adapts VPGNet [3] four-quadrant vanishing point localization method. This approach partitions the image plane using quadrant masks, defining the vanishing point (VP) as the intersection of these four regions. Through this structural decomposition, the vanishing point prediction (VPP) module infers the VP position by analyzing quadrant-specific features within the segmented global scene structure.

OTVLD-Net uses a lightweight semantic segmentation module in the vanishing point prediction head, which contains a 6 × 6 residual block, two 1 × 1 residual blocks, and finally performs a Tiling upsampling operation on the obtained feature map to obtain a four-quadrant vanishing point mask map with 5 channels. In the four-quadrant vanishing point mask map, VP is the intersection of the four quadrants, so the confidence values of VP in the four quadrant channels are roughly the same. Based on this, the formula for calculating VP is as follows:

(5) $$P_{avg}=\frac{1-(\sum p_{0}(x,y))}{4mn}$$

(6) $$loc_{vp}=\underset{\left(x,y\right)}{argmin}\sum_{n=1}^{4}{\left|P_{avg}-p_{n}(x,y)\right|}^{2}$$

Among them, $P_{avg}$ represents the probability of the existence of VP in the image; $p_{n}(x,y)$ represents the confidence of the point (x,y) in n quadrant channel, m×n represents the size of the confidence map, and $loc_{vp}$ represents the position coordinates of the final predicted VP.

In OTVLD-Net, the VPP module serves as an auxiliary component exclusively during training to enhance spatial perception. This module is omitted during inference to optimize computational efficiency.

### 3.4. Transformer Global Feature Aggregation

**Figure 3:**
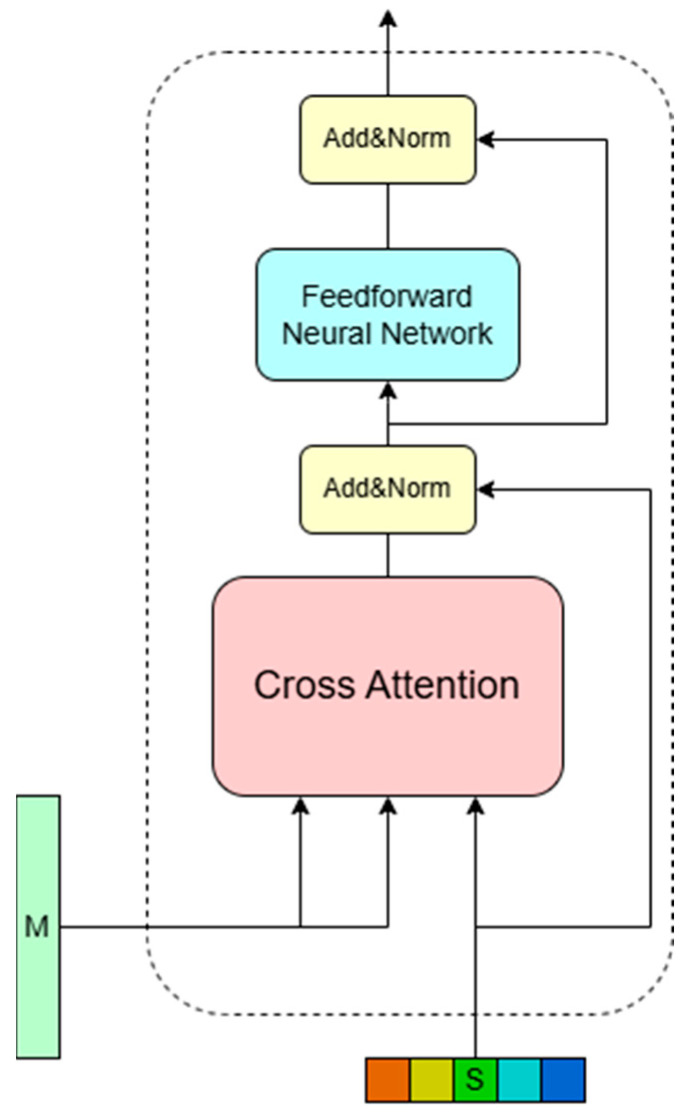
Lane feature cross-attention decoder.

To generate dynamic feature weights for each lane line candidate, the Transformer global feature aggregation module is designed in this paper, as shown in Figure 3. The encoder of the model takes the feature sequence I as input, uses the Transformer self-attention mechanism to capture each of the most relevant input features in I, and outputs the feature sequence $m\in R^{HW\times C}$. However, this module discards the mask-self-attention module in a typical Transformer decoder, and defines a learnable lane query sequence $s\in R^{L\times C}$, where L vectors of length C represent different lane line candidates, and this query sequence s will be directly used as the query sequence q of the decoder’s self-attention module after a linear transformation. At this time, the decoder self-attention module integrates the cross-attention mechanism to capture the most relevant features from the feature sequence m for each lane line query in s. Finally, the decoder will output the dynamic lane feature sequence $t\in R^{L\times C}$. The functional relationship between the query sequence q and the feature sequence m in the cross-self-attention mechanism is as follows:

(7) $$A_{i,j}=\frac{\exp(q_{i}^{T}k_{j})}{\sum_{l=1}^{HW}\exp(q_{i}^{T}k_{l})}$$

(8) $$t_{i}=g(\sum_{j=i}^{HW}A_{i,j}v_{j})$$

Among them, k and v represent the key vector and value vector obtained by linear transformation of m, respectively, $q_{i}$ and $k_{i}$ represent the *i*-th and *j*-th unknown features of q and k, respectively, A is used to describe the attention map of the pairwise relationship $A_{i,j}$ between $q_{i}$ and $k_{i}$, $t_{i}$ represents the lane feature of i output corresponding to i lane query $S_{i}$, and g() is a nonlinear mapping.

Within this architecture, the dynamic lane feature sequence t is generated through cross-attention between the lane query sequence s and the globally enhanced pixel feature sequence m. Consequently, each lane feature $t_{i}$ effectively captures channel-wise global context for its corresponding lane in $s_{i}$. When integrated with spatial global features from Non-Local Blocks in the feature extraction module, OTVLD-Net achieves comprehensive global perception across dimensions. This enables precise localization of image pixels most relevant to lane queries $s_{i}$ demonstrating robustness against challenging conditions including occlusions and complex topological variations.

### 3.5. Lane Detection Module

To generate pixel-level lane predictions, the model leverages dynamically generated weights from preceding modules within its detection pipeline. At this stage, the model reshapes the output feature sequence $m\in R^{HW\times C}$ of the Transformer encoder into a feature map $M^{\prime }\in R^{H\times W\times C}$, multiplies it by the heat map kernel $K_{b}\in R^{L\times C}$ and the offset kernel $K_{z}\in R^{L\times C}$, and obtains the lane line pixel point heat map $B\in R^{H\times W\times L}$ and the offset map $Z\in R^{H\times W\times L}$. Then the model will use the inter-row softmax to further process the lane line pixel point heat map B:

(9) $$B_{ijk}=\frac{\exp(B_{ijk})}{\sum_{m=1}^{W-1}B_{ijm}}$$

Among them, $B_{ijk}$ represents the probability that i predicted lane line exists in j row and k column of the lane line pixel point heat map B. For simplicity, the same name B will be used to represent the above results in the following.

Each lane feature $T_{i}$ corresponds to a heat map $B_{i}\in R^{H\times W}$ and an offset map $Z_{i}\in R^{H\times W}$. The heat map B predicts the probability of each pixel being a lane point (foreground) for each predicted lane line, and the offset map Z predicts the horizontal offset from the lane point of each pixel in the same row to each predicted lane line (at this time, it is assumed that each $T_{i}$ has at most one lane pixel in each row). After the model inputs the lane line pixel heat map and offset map into a post-processing step, it combines the vertical range vector $v\in R^{L\times C}$ and the object score vector $c\in R^{L\times 2}$ to obtain the final lane line pixel point. This post-processing process is shown in the following formula:

(10) $$E=\sum_{k=0}^{W-1}B_{ijk}\cdot k$$

(11) $$P_{ij}=(E_{ij}+Z_{ijE_{ij}},j)$$

where E represents the expected horizontal coordinate of each lane line in each row generated initially using the lane line pixel point heat map B; P represents the resultant horizontal coordinate of each lane line in each row obtained by combining the expected E with the position offset of each lane line in the lane line pixel point offset map Z. The calculation process of the final model predicting the lane line point is as follows:

(12) $$L_{i}=\left\{P_{ij}|v_{i0}\leq j\leq v_{i1}\right\}$$

(13) $$Y=\left\{L_{i}|c_{i1}\geq t\right\}$$

where $L_{i}$ represents the point set of i lane line predicted by the model, among which only the lane points between the starting row $V_{i0}$ and the ending row $V_{i1}$ are retained; Y represents the set of lane lines finally predicted by the model, among which only the lane lines with foreground probability $C_{i1}$ higher than the threshold t are retained.

### 3.6. Joint Loss Function

The proposed OTVLD-Net employs joint training for its two complementary subtasks: lane detection and vanishing point detection. Accordingly, we design a joint loss function to evaluate model performance and optimize parameters. The overall loss function for lane detection in OTVLD-Net is formulated as:

(14) $$L_{lane\_detection}=\lambda_{vpp}L_{vpp}+\lambda_{bipartite\_match}L_{bipartite\_match}$$

The vanishing point prediction VPP loss calculates the loss of VPP prediction by calculating the Euclidean distance between the predicted VP and the true value VP. By converting the distance between the predicted point and the true value point into a probability distribution, assuming that the predicted point is $\hat{y}$ and the true value point is y, their Euclidean distance in two-dimensional space is $d(\hat{y},y)$. VPP loss uses a Gaussian distribution, sets a standard deviation σ to convert the square of the Euclidean distance into a probability density function of a Gaussian distribution, and uses the negative log-likelihood loss as the loss function for the result:

(15) $$L_{VPP}=\log(2\pi \sigma^{2})+\frac{1}{\sigma^{2}}d(\hat{y},y{)}^{2}$$

In the lane detection stage, the final predicted lane pixel set L, lane pixel heat map B, offset map Z, vertical range vector v, and object score vector c are generated. First, the model will calculate the pairwise bipartite matching loss $L_{match}(Y_{i},Y_{j}^{*})$ of L predicted lane lines $Y={\left\{Y_{i}\right\}}_{i=1}^{L}$ and M true lane lines $Y^{*}={\left\{{Y_{i}}^{*}\right\}}_{i=1}^{M}$, which is expressed as the weighted sum of the object score loss $L_{obj}(Y_{i},Y_{j}^{*})$, lane pixel heat map loss $L_{heat}(Y_{i},Y_{j}^{*})$, lane pixel offset map loss $L_{off}(Y_{i},Y_{j}^{*})$, and vertical range loss $L_{rng}(Y_{i},Y_{j}^{*})$. Among them, the calculation process of the object score loss $L_{obj}(Y_{i},Y_{j}^{*})$ is:

(16) $$L_{obj}(Y_{i},Y_{j}^{*})=-\log(c_{i1})$$

where $C_{i1}$ represents the probability that the *i*-th lane line is the foreground lane line. The calculation process of the lane line pixel heat map loss $L_{heat}(Y_{i},Y_{j}^{*})$ is:

(17) $$L_{heat}(Y_{i},Y_{j}^{*})=\frac{1}{R_{j1}^{*}-R_{j0}^{*}}\sum_{k=R_{j0}^{*}}^{R_{j1}^{*}}\left\|\sum_{m=0}^{W-1}B_{ikm}\cdot m-P_{jk0}^{*}\right\|$$

where $B_{ikm}$ represents the probability that the pixel point of i lane line in k row and m column is a foreground pixel point; $P_{jk0}^{*}$ represents the horizontal coordinate of j true value lane line in k row; and $R_{i1}^{*}$ and $R_{i0}^{*}$ represent the vertical coordinates of the starting point and the ending point of true value lane line, that is, the pixel point heat map loss is only calculated within the valid range of the true value lane. The calculation process of the lane line pixel offset map loss $L_{off}(Y_{i},Y_{j}^{*})$ is:

(18) $$L_{off}(Y_{i},Y_{j}^{*})=\frac{1}{W(R_{j1}^{*}-R_{j0}^{*})}\sum_{k=R_{j0}^{*}}^{R_{j1}^{*}}\sum_{m=0}^{W-1}\left\|Z_{ikm}+m-P_{jk0}^{*}\right\|$$

where $Z_{ikm}$ represents the predicted horizontal offset of the pixel at k row and m column of i lane line; $Z_{ikm}+m$ represents the horizontal coordinate of i lane line predicted by the model at k row; the pixel offset map loss is the same as the pixel point heat map loss, which is only calculated within the valid range of the true value lane. The calculation process of the vertical range loss $L_{rng}(Y_{i},Y_{j}^{*})$ is:

(19) $$L_{rng}(Y_{i},Y_{j}^{*})=\left\|R_{i0}-R_{j0}^{*}\right\|+\left\|R_{i1}-R_{j1}^{*}\right\|$$

where $R_{i0}$ and $R_{i1}$ represent the predicted starting and ending ordinates of k predicted lane line. The calculation process of the final lane line bipartite matching loss is:

(20) $$L_{match}(Y_{i},Y_{j}^{*})=\lambda_{obj}L_{obj}(Y_{i},Y_{j}^{*})+\lambda_{heat}L_{heat}(Y_{i},Y_{j}^{*})+\lambda_{off}L_{off}(Y_{i},Y_{j}^{*})+\lambda_{rng}L_{rng}(Y_{i},Y_{j}^{*})$$

The $\lambda_{obj}$, $\lambda_{heat}$, $\lambda_{off}$, and $\lambda_{rng}$ are weighted balance coefficients of $L_{obj}$, $L_{heat}$, $L_{off}$, and $L_{rng}$, respectively. Subsequently, the model will use a mapping function z(j) to represent the optimal prediction-true value match, that is, the index of the predicted lane line assigned to j true value lane line in the optimal match. The minimum matching loss guided by it is obtained:

(21) $$z(j)=\arg\underset{z}{min}\hat{z}=\sum_{j=1}^{N}L_{match}(y_{z(j)},y_{j}^{*})$$

After obtaining the optimal match z(j) between the predicted value and the true value, the calculation process of the final lane line two-part matching loss function is as follows:

(22) $$L_{bipartite_{match}}=\frac{1}{N}\sum_{j=1}^{N}\left[\lambda_{obj}L_{obj}\left(Y_{i},Y_{j}^{*}\right)+\lambda_{heat}L_{heat}\left(Y_{i},Y_{j}^{*}\right)+\lambda_{off}L_{off}\left(Y_{i},Y_{j}^{*}\right)+\;\lambda_{rng}L_{rng}\left(Y_{i},Y_{j}^{*}\right)\right]+\frac{1}{L-N}\sum_{i\notin v}\lambda_{obj}L_{obj}\left(Y_{i},\phi \right)$$

where $L_{obj}(Y_{i},\phi )=-\log(C_{i0})$, $C_{i0}$ represents the probability that the *i*-th predicted lane line is the background.

## 4. Experimental and Results

### 4.1. Lane Line Dataset

To comprehensively evaluate the proposed method and validate its efficacy, we conduct experiments on two benchmark lane detection datasets: OpenLane [28] and CurveLanes [29]. OpenLane comprises 160,000 training images and 40,000 validation images across six challenging scenarios: curves, intersections, nighttime conditions, extreme weather, merging/splitting lanes, and uphill/downhill terrain. The dataset features annotations for 14 lane categories, including road edges and double yellow solid lines. CurveLanes contains 100,000 training, 20,000 validation, and 30,000 test images, with extensive coverage of complex scenarios such as high-curvature roads, bifurcations, and dense lane configurations.

### 4.2. Evaluation Indicators

Both OpenLane and CurveLanes datasets employ the F1 score as the primary evaluation metric. True positives (TP), false positives (FP), and false negatives (FN) are determined by computing the Intersection-over-Union (IoU) between predicted lane markings and ground truth annotations. The IoU of two lane lines is defined as the IoU between their masks, where the width of the mask is fixed to 30 pixels. The F1 score is calculated as follows:

(23) $$Precison=\frac{N_{TP}}{N_{TP}+N_{FP}}$$

(24) $$Recall=\frac{N_{TP}}{N_{TP}+N_{FN}}$$

(25) $$F1=\frac{2\times Precision\times Recall}{Precision+Recall}$$

### 4.3. Experimental Setup

To ensure the accuracy and stability of the experiment, the following configuration was used: GPU is NVIDIA GeForce RTX3080Ti, single card is used. Python version is 3.8.13, NVCC version is Cuda compilation tool, version 11.7, V11.7.99. GCC version is gcc 9.4.0. PyTorch version is 1.13.0, CuDNN version is 8.5. OpenCV version is 4.2.0.

In terms of model structure, the number of lane line queries L is set to 80 to cover the number of all possible lane lines in a complex road scene. Meanwhile, in order to strike a balance between model performance and computational efficiency, the number of layers of the Transformer encoder and decoder are set to 2 and 4, respectively. In the vanishing point detection loss, the initial standard deviation σ is set to 0.5, and when the model is trained to the 30th epoch, the standard deviation σ is adjusted to 0.1 in order to prevent the gradient from decreasing too fast or oscillating, and in the two-part lane line matching loss, in order to make the individual loss terms consistent in order to promote the model to be consistent in terms of magnitude. In the experiments, the target detection loss weight $\lambda_{obj}$ is set to 5, the heat map loss weight $\lambda_{heat}$ is set to 1, the offset loss weight $\lambda_{off}$ is set to 1, and the range loss weight $\lambda_{rng}$ is set to 10. In the post-processing stage, the target score threshold t is set to 0.7 to filter out the lane lines with higher confidence. Lane line prediction results with higher confidence.

### 4.4. Comparative Experiment

The performance evaluation results on the OpenLane dataset are shown in Table 1 OTVLD-Net achieved F1 scores of 59.8%, 61.2%, and 62.5% when using ResNet-18, ResNet-34, and ResNet-101 as the template feature extraction networks, respectively, outperforming the best-performing experimental one, which used ResNet- 101 as the backbone network for CondLaneNet, with corresponding improvements of 1.9%, 4.2%, and 6.4%, respectively. In different challenging lane scenarios, OTVLD-Net achieved the best F1 scores, as shown in Table 2, demonstrating the robustness of the model. In particular, in the case of the feature extraction network using ResNet-18 as a template, OTVLD-Net achieves F1 scores of 63.9%, 54.5%, and 62.5% in the “curves”, “intersections”, and “merging and diverging” scenarios, respectively, which are 6.4%, 6.1%, and 17.0% better than CondLaneNet, respectively. These results show that the OTVLD-Net architecture can well handle lane lines with complex topologies. The reason is that by using ODVT-Net, a full-dimensional dynamic nonlocal fusion feature extraction network, the model is able to dynamically adjust the extraction weights of features according to different lane line scenarios, and the hybrid attention mechanism of nonlocal non-local combined with Transformer is able to capture the rich global information of lane lines from the feature maps across space and dimension, which makes it better able to distinguish different lane lines than other methods that do not use the attention mechanism or only combine channel or spatial attention. The hybrid attention mechanism can capture the rich global information of lane lines from the feature map across space and dimensions, making it better able to distinguish different lane lines compared to other methods that do not use an attention mechanism or only combine channel or spatial attention. In terms of speed, the feature extraction network (ODVT-Net-18) using ResNet-18 as the template achieves 103 FPS and 14.2 GFlops of computation with an F1 score of 59.8%, which is a good performance while maintaining real-time performance.

**Figure 4:**
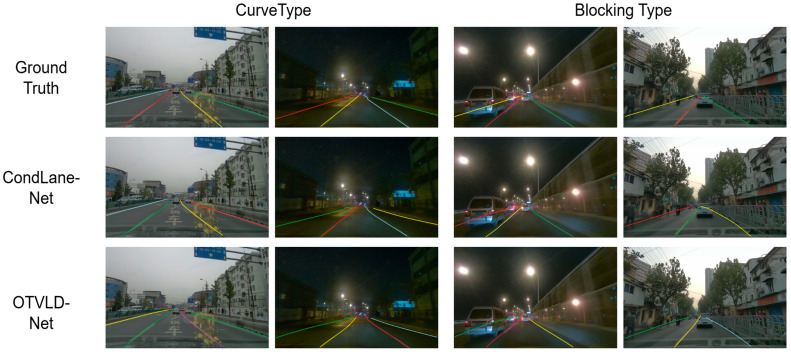
Qualitative comparison result 1 on different challenging scenarios of the CurveLanes dataset.

**Figure 5:**
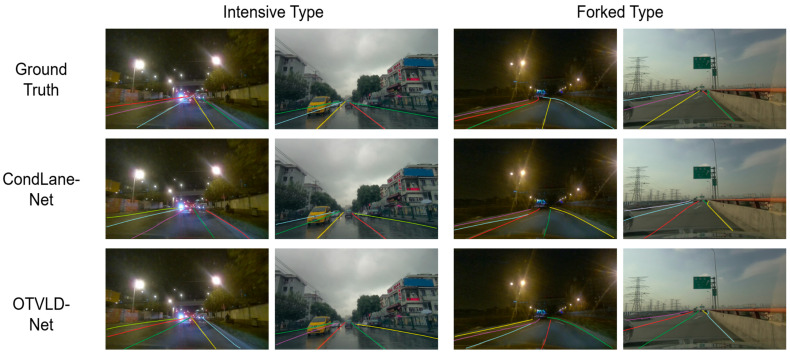
Qualitative comparison result 2 on different challenging scenarios of the CurveLanes dataset.

The performance on the CurveLanes dataset is shown in Table 3. The CurveLanes dataset contains lane lines with complex topologies and occlusion phenomena, such as curved, bifurcated, dense, and blocked lane lines. Using ResNet-18, ResNet-34, and ResNet-101 as templates for the feature extraction network, OTVLD-Net achieved F1 scores of 87.86%, 88.16%, and 88.35%, respectively, and improved 2.90%, 2.51%, and 2.38%, respectively, over CondLaneNet, which had the next highest performance. Meanwhile, some qualitative comparison results on the CurveLanes dataset, which are classified into four categories: curvilinear, blocked, dense, and bifurcated lane lines, are presented in Figure 4 and Figure 5 The experimental results show that OTVLD-Net is able to effectively deal with the obstruction problem as well as lane lines with complex topologies.

### 4.5. Ablation Experiment

To evaluate the impact of the Feature Flip Fusion module on model performance, we conducted an ablation study while maintaining identical model architecture elsewhere. As shown in Table 4, incorporating the Feature Flip Fusion module yields a substantial 4.34% improvement in F1 score. This enhancement demonstrates that the module’s exploitation of inherent lane symmetry provides complementary information critical for lane detection accuracy.

To validate the efficacy of the ODVT-Net feature extraction network, we employ ODVT-Net-34—a ResNet-34 variant embedding ODConv and Non-Local layers—for comparative evaluation against standard backbone networks. Ablation results in Table 5 demonstrate that ODVT-Net exhibits significantly enhanced dynamic adaptation capabilities while effectively leveraging spatial global context to deliver superior feature representations for subsequent lane detection modules.

To investigate the effect of varying the number of lane queries S on model performance, the experimental results are presented in Table 6 Increasing S from 20 to 80 enhances the model ability to capture and represent lane information characterized by diverse topologies. However, further increasing S introduces excessive redundancy, which adversely affects further performance improvement.

To investigate the effect of varying the number of decoder layers on model performance, experimental results are presented in Table 7. Increasing the number of decoder layers from 1 to 4 enhances the dynamic convolutional kernel’s capacity to capture global lane line information, thereby improving detection accuracy. However, further increasing the number of layers yields diminishing returns for global information capture due to introduced computational redundancy, with no significant performance improvement observed.

In order to explore the impact of the vanishing point prediction module on the model performance, the ablation experiment conducted in this section is shown in Table 8. While keeping the other structures of the model the same, the experimental results are compared and found that after the introduction of the vanishing point prediction module, the F1 score of the model reaches 88.23%, which is 4.07% higher than that without the introduction of the module. This result shows that the global geometric prior information provided by the vanishing point prediction module can significantly enhance the robustness of lane line detection. Especially in curved roads, occluded scenes, and low light conditions, helping the model to correct local perception bias. The addition of the vanishing point prediction module enables the model to utilize the topological characteristics of the scene depth to enhance the geometric reasoning ability of the lane line direction, and ultimately improves the detection accuracy in complex driving environments.

In order to explore the impact of the lane detection module on the model performance, the ablation experiments conducted in this section are shown in Table 9. While keeping the other structures of the model unchanged, the F1 score is improved by 5.35% after introducing the designed lane detection head. This result shows that the design and performance of the lane detection module are crucial to accurately decode features into precise lane line predictions. The proposed detection head significantly enhances the model’s ability to aggregate local features and resolve spatial ambiguity, especially in challenging scenarios involving severe perspective distortion, long-distance lane lines, or dense adjacent lanes. The detection module designed in this paper is crucial in maintaining detection integrity and suppressing false positives.

To verify whether the proposed Omni-Dimensional Dynamic Non-Local Fusion Feature Extraction Network (ODVT-Net) and Transformer Global Feature Aggregation (TGFA) can improve lane detection performance, we designed different combinations of the three feature fusion modules, ODVT-Net, TGFA, RESA [30], and SCNN, and analyzed them on the CULane dataset. The quantitative results are shown in Table 10 below. The results show that for the backbone network, ODVT-Net-34 leads the residual network in accuracy, while significantly reducing the number of parameters and inference time, verifying that its application-side matching is higher than that of the residual network; for the feature aggregation module, while maintaining the detection accuracy and number of parameters, the inference time of the Transformer global feature aggregation module is relatively slow, but the gap is not large, thus verifying the superiority of the newly designed module in this chapter in the lane line detection task.

## 5. Conclusions

This paper proposes a lane detection model OTVLD-Net based on full-dimensional dynamic convolutional Transformer. In the feature extraction stage, the model designs a full-dimensional dynamic non-local feature extraction network ODVT-Net, which dynamically adapts to different types of complex lane inputs through ODConv, and combines Non-Local Level and Feature Flip Fusion Level to obtain richer contextual information using the horizontal symmetry of the lane. While using the self-attention mechanism of the Transformer encoder, a cross-attention mechanism between the feature map and the lane request is added in the decoder stage, and a feature weight generation mechanism based on Transformer is designed, which can intuitively capture the correlation between the feature sequence and the lane prior sequence and reduce the network complexity. The vanishing point detection module is introduced to assist the lane detection task, and a weighted loss function consisting of bipartite matching loss and vanishing point detection is designed. The model is trained together with the vanishing point detection to share weights, which enhances the generalization ability of the model. The performance of OTVPA-Net on the OpenLane dataset is 6.4% higher than that of the second-ranked model. At the same time, the detection speed of OTVPA-Net reached 103 FPS, the computing amount was only 14.2 GFlops, and the real-time performance was greatly improved. The performance and design of the model were verified through a series of comparative experiments and ablation experiments. However, the vanishing point detection module relies on the geometric structure of the image plane and has poor robustness in low-visibility scenarios such as extreme rainstorms/heavy fog, as well as in severely occluded environments. Furthermore, the model only outputs two-dimensional lane line pixel positions and lacks three-dimensional geometric information such as lane line height and curvature radius, making it unable to meet the requirements for lane spatial topology (such as uphill/curved curvature) in actual driving. Training relies on the OpenLane/CurveLanes dataset and does not cover unstructured roads. Therefore, in order to address the shortcomings of this article, in the future we will integrate radar/lidar point cloud data to compensate for the lack of vision in low visibility, refer to mainstream 3D lane line detection and BEV lane maps for 3D perception, and compare with the RSUD20K dataset to verify the generalization ability of roads in developing countries (such as narrow streets in Bangladesh and mixed rickshaw traffic).

## Figures and Tables

**Table 1:** Performance evaluation results on the OpenLane dataset.

Model	Backbone	F1	FPS	GFlops
SCNN	VGG-16	0.175	143	11.2
Enet-SAD	ENet	0.181	135	12.1
PointLaneNet	GoogleNet	0.197	126	13.5
LaneATT	ResNet-18	0.282	151	**9.5**
LaneATT	ResNet-34	0.315	127	18.2
PersFormer [11]	EfficientNet-B7	0.418	106	20.4
LSTR [27]	ResNet-18	0.406	103	18.9
CLRNet [29]	ResNet-34	0.575	130	20.1
CurveLane	Searched-S	0.582	99	13.7
CurveLane	Searched-MM	0.603	90	21.5
CurveLane	Searched-L	0.614	42	47.9
CondLaneNet	ResNet-18	0.532	**172**	10.4
CondLaneNet	ResNet-34	0.546	126	19.8
CondLaneNet	ResNet-101	0.587	45	45
**OTVLD-Net**	ODVT-Net-18	0.598	103	14.2
**OTVLD-Net**	ODVT-Net-34	0.612	93	23.4
**OTVLD-Net**	ODVT-Net-101	**0.625**	43	50.4

**Table 2:** Performance evaluation results on different challenging lane scenarios in the OpenLane dataset.

Model	Backbone	Uphill and Downhill	Bend	Extreme Weather	Night Road	Intersection	Convergence and Divergence
SCNN	VGG-16	0.135	0.121	0.198	0.132	0.092	0.137
Enet-SAD	ENet	0.156	0.143	0.236	0.161	0.113	0.167
PointLaneNet	GoogLeNet	0.189	0.217	0.282	0.193	0.129	0.203
LaneATT	ResNet-18	0.252	0.256	0.318	0.274	0.138	0.241
LaneATT	ResNet-34	0.281	0.272	0.345	0.319	0.168	0.263
PersFormer	EfficientNet-B7	0.405	0.461	0.435	0.359	0.287	0.410
LSTR	ResNet-18	0.392	0.439	0.421	0.313	0.265	0.407
CLRNet	ResNet-34	0.611	0.625	0.533	0.492	0.536	0.519
CurveLane	Searched-S	0.521	0.597	0.492	0.483	0.517	0.606
CurveLane	Searched-MM	0.577	0.634	0.511	0.509	0.549	0.614
CurveLane	Searched-L	0.602	0.669	0.525	0.543	0.561	0.635
CondLaneNet	ResNet-18	0.552	0.573	0.456	0.464	0.482	0.453
CondLaneNet	ResNet-34	0.583	0.591	0.490	0.484	0.505	0.476
CondLaneNet	ResNet-101	0.619	0.627	0.545	0.508	0.555	0.521
**OTVLD-Net**	ODVT-Net-18	0.560	0.637	0.513	0.509	0.543	0.623
**OTVLD-Net**	ODVT-Net-34	0.589	0.652	0.532	0.539	0.571	0.630
**OTVLD-Net**	ODVT-Net-101	**0.620**	**0.688**	**0.549**	**0.571**	**0.583**	**0.656**

**Table 3:** Performance evaluation results on the CurveLanes dataset.

Model	Backbone	F1	Accuracy	Recall
SCNN	VGG-16	0.6496	0.7621	0.5661
Enet-SAD	Enet	0.5017	0.6348	0.4148
PointLaneNet	GoogleNet	0.7893	0.8621	0.7279
LaneATT	ResNet-18	0.7982	0.8683	0.7305
LaneATT	ResNet-34	0.8089	0.8725	0.7481
PersFormer	EfficientNet-B7	0.8132	0.8753	0.8115
LSTR	ResNet-18	0.8212	0.8791	0.8226
CLRNet	ResNet-34	0.8289	0.8832	0.8297
CurveLane	Searched-S	0.8099	**0.9345**	0.7146
CurveLane	Searched-MM	0.8167	0.9336	0.7258
CurveLane	Searched-L	0.8219	0.9104	0.7490
CondLaneNet	ResNet-18	0.8496	0.8762	0.8245
CondLaneNet	ResNet-34	0.8565	0.8816	0.8328
CondLaneNet	ResNet-101	0.8597	0.8885	0.8327
**OTVLD-Net**	ODVT-Net-18	0.8786	0.9077	0.8514
**OTVLD-Net**	ODVT-Net-34	0.8816	0.9112	0.8539
**OTVLD-Net**	ODVT-Net-101	**0.8835**	0.9120	**0.8568**

**Table 4:** Quantitative evaluation of whether to use the feature flipping fusion module on the CurveLanes dataset.

Whether to Add Feature Flip Fusion Module	F1	Accuracy	Recall
No	0.8452	0.8977	0.8458
Yes	**0.8819**	**0.9087**	**0.8514**

**Table 5:** Quantitative evaluation of feature extraction networks on the CurveLanes dataset.

Backbone	F1	Accuracy	Recall
VGG-16	0.6728	0.7984	0.5841
SENet	0.7243	0.8465	0.6428
EfficientNet-B7	0.7756	0.8926	0.7085
ResNet-34	0.7924	0.9033	0.7859
ODVT-Net-34	**0.8816**	**0.9112**	**0.8539**

**Table 6:** Quantitative evaluation of the number of lane queries S on the CurveLanes dataset.

Lane Query Number	F1	Accuracy	Recall
20	0.8725	0.8917	**0.8541**
40	0.8746	0.8976	0.8532
80	**0.8789**	0.9078	0.8514
100	0.8787	**0.9108**	0.8497

**Table 7:** Quantitative evaluation of the number of decoders on the CurveLanes dataset.

Number of Decoders	F1	Accuracy	Recall
1	0.8671	0.8963	0.8396
2	0.8710	0.8997	0.8439
4	0.8788	0.9076	0.8513
6	**0.8804**	**0.9105**	**0.8531**

**Table 8:** Quantitatively evaluate whether to use the vanishing point prediction module on the CurveLanes dataset.

Whether to Add VPP Module	F1	Accuracy	Recall
No	0.8478	0.8926	0.8473
Yes	**0.8823**	**0.9032**	**0.8554**

**Table 9:** Quantitatively evaluate whether to use the lane detection module on the CurveLanes dataset.

Whether to Add Lane Detection Module	F1	Accuracy	Recall
No	0.8361	0.8872	0.8353
Yes	**0.8809**	**0.8997**	**0.8523**

**Table 10:** The impact of model components on model performance on the CurveLanes dataset.

Module Components	F1	Params/M	Inference Time/ms
ResNet-18+RESA	0.7471	16.25	15
ResNet-18+SCNN	0.6942	14.83	9
ResNet-18+TGFA	0.7519	13.12	12
ODVT-Net-34+RESA	0.8593	12.9	9
ODVT-Net-34+SCNN	0.8436	11.24	**7**
ODVT-Net-34+TGFA	**0.8816**	**10.06**	8

## Data Availability

The data presented in this study are available upon request from the corresponding author.
